# Chemical-genetic induction of Malonyl-CoA decarboxylase in skeletal muscle

**DOI:** 10.1186/1471-2091-15-20

**Published:** 2014-08-25

**Authors:** Susana Rodriguez, Jessica M Ellis, Michael J Wolfgang

**Affiliations:** 1 Department of Biological Chemistry, Center for Metabolism and Obesity Research, Johns Hopkins University School of Medicine, 725 N. Wolfe St., 475 Rangos Building, Baltimore, Maryland 21205, USA

**Keywords:** Diabetes, Metabolism, Insulin resistance, Fatty acid oxidation, Chemical-genetics

## Abstract

**Background:**

Defects in skeletal muscle fatty acid oxidation have been implicated in the etiology of insulin resistance. Malonyl-CoA decarboxylase (MCD) has been a target of investigation because it reduces the concentration of malonyl-CoA, a metabolite that inhibits fatty acid oxidation. The *in vivo* role of muscle MCD expression in the development of insulin resistance remains unclear.

**Results:**

To determine the role of MCD in skeletal muscle of diet induced obese and insulin resistant mouse models we generated mice expressing a muscle specific transgene for MCD (Tg-fMCD^Skel^) stabilized posttranslationally by the small molecule, Shield-1. Tg-fMCD^Skel^ and control mice were placed on either a high fat or low fat diet for 3.5 months. Obese and glucose intolerant as well as lean control Tg-fMCD^Skel^ and nontransgenic control mice were treated with Shield-1 and changes in their body weight and insulin sensitivity were determined upon induction of MCD. Inducing MCD activity >5-fold in skeletal muscle over two weeks did not alter body weight or glucose intolerance of obese mice. MCD induction further potentiated the defects in insulin signaling of obese mice. In addition, key enzymes in fatty acid oxidation were suppressed following MCD induction.

**Conclusion:**

Acute induction of MCD in the skeletal muscle of obese and glucose intolerant mice did not improve body weight and decreased insulin sensitivity compared to obese nontransgenic controls. Induction of MCD in skeletal muscle resulted in a suppression of mitochondrial oxidative genes suggesting a redundant and metabolite driven regulation of gene expression.

## Background

The concomitant rise in obesity and type 2 diabetes has mustered a global effort to understand the links between nutrient overload and insulin resistance to enable new therapies. The skeletal muscle plays an important role in maintaining systemic glycemic control by mediating a majority of insulin stimulated glucose disposal. Skeletal muscle has been demonstrated to be a primary tissue driving insulin resistance and is the target for several anti-diabetic drugs [1-3]. Excess lipid accumulation outside of adipose tissue is thought to contribute to diabetes by engaging pathways that inhibit insulin signaling. The mechanisms that lead to the development of lipid-induced insulin resistance remain elusive, but consistent themes converge at pathways implicating inflammation, ER stress, and mitochondrial insufficiency [4-7].

Skeletal muscle with its high capacity for fatty acid oxidation has been a target for genetic and pharmacological studies intended to restore lipid balance by promoting lipid oxidative pathways. From these studies, multiple mechanisms have been proposed to connect lipid metabolism and defects in insulin sensitivity. For example, mitochondria are the major site for fatty acid oxidation and defects in this process may contribute to lipotoxic pathways. The lipotoxicity hypothesis suggests that accumulation of lipid signaling intermediates interact and disrupt insulin signaling to mediate or exacerbate insulin resistance [8-12]. The finding that the muscles of patients with type 2 diabetes contained fewer and smaller mitochondria than those of age matched insulin sensitive controls, further supported the concept that mitochondrial deficiency or dysfunction is a driver of insulin resistance [13-19]. The muscle’s decreased mitochondrial content limits its capacity to oxidize fatty acids, resulting in the accumulation of undesirable intramuscular lipids, such as ceramide and DAG [20,21]. Therefore, methods that increase fatty acid oxidation, akin to exercise, in the muscle to relieve the toxicity caused by these lipid intermediates have been sought to improve insulin resistance.

Malonyl-CoA is the substrate for *de novo* fatty acid synthesis and its concentration is dependent on the nutritional status of the cell. Malonyl-CoA is produced by acetyl-CoA carboxylase (ACC) and catabolized by malonyl-CoA decarboxylase (MLYCD, commonly referred to as MCD) in the cytoplasm [22]. Although malonyl-CoA is the substrate for fatty acid synthase (FAS) for the production of fatty acids *de novo*, FAS is not expressed at high levels in skeletal muscle [23,24]. In the skeletal muscle, the primary role of MCD is to decarboxylate malonyl-CoA thereby enhancing fatty acid oxidation by alleviating the allosteric inhibition of malonyl-CoA on the rate-setting step in mitochondrial long chain fatty acid beta-oxidation, Carnitine Palmitoyltransferase 1 (CPT1). Genetic and pharmacological studies to inhibit or activate genes involved in fatty acid synthesis (ACC2) or oxidation (CPT1, MCD, AMPK) have produced conflicting results supporting the benefits of skeletal muscle mitochondrial fatty acid oxidation in models of diet induced insulin resistance [5,25-31]. Whole body deletion of ACC2, used to promote fat oxidation by decreasing malonyl-CoA levels, produced lean hyperphagic mice that exhibited increased fatty acid oxidation, increased energy expenditure, and improved insulin sensitivity [32]. The potential benefits from decreased malonyl-CoA levels to promote fatty acid oxidation to reduce body mass and increase insulin sensitivity prompted the development of other models of ACC2 deletion. New models of whole body and skeletal muscle ACC2 deletion or pharmacological inhibition of ACC2, exhibited no protection from obesity and insulin resistance, while energy expenditure remained unaffected [25,26].

MCD, an enzyme that promotes fatty acid oxidation, has been used to elucidate the relationship between glucose and lipid oxidation in the development of insulin resistance. Over-expression of MCD in the liver of rats fed a high fat diet was shown to increase fatty acid oxidation and improve whole body insulin resistance [31]. Conversely, the loss of whole body MCD resulted in resistance to diet-induced glucose intolerance, despite high intramuscular levels of triacylglycerol and long chain fatty acids [33]. A study using human cultured skeletal myotubes investigated the effects of shifting substrate oxidation from lipid to glucose via RNA interference-mediated gene silencing of MCD under basal and insulin-stimulated conditions [28]. Reducing MCD expression in human myotubes, led to decreased lipid oxidation of palmitate with a rise in glucose oxidation under insulin stimulation. However, several of these studies are confounded by the loss of both cytosolic and mitochondrial MCD, which is implicated in the clearance of mitochondrial malonate, a cytotoxic metabolite that inhibits succinate dehydrogenase [34-39].

The interconnective nature of metabolic pathways, coupled with the redundancy and compensatory effects often seen by over-expression and knockout studies make it difficult to tease out the contributions of individual pathways to the pathophysiology of insulin resistance in skeletal muscle. Chemical-genetic techniques have been developed to acutely alter metabolic pathways in a manner that is temporal, cell-specific, and reversible [40-42]. We have previously shown the posttranslational regulation of MCD in transgenic mice in a tissue specific manner via a biologically inert small molecule, Sheild-1 [40]. The benefits of this methodology over previous models are the ability to control for off target effects of the chemical in nontransgenic controls and the ability to alter metabolic pathways in already pathogenic animal models. Here, we acutely induced MCD in the skeletal muscle of obese and glucose intolerant mice to determine the impact of modulating skeletal muscle fatty acid oxidation in a model of diet-induced obesity. Surprisingly, a two week induction of MCD in skeletal muscle did not alter body weight or ameliorate glucose intolerance, conversely it further impaired insulin signaling in the skeletal muscle of diet-induced obese mice. Furthermore, an acute induction of MCD led to a suppression of fatty acid oxidative genes suggesting a redundant and metabolite driven regulation of gene expression.

## Methods

### Antibodies and chemicals

Rabbit polyclonal antibodies that recognize phospho-AKT (Ser473), Pan AKT, phospho-GSK3b (Ser9), Pan GSK3b, phospho-glycogen synthase (Ser 641), Pan glycogen synthase, phospho-IRS-1 (Ser 302), Pan IRS-1, phospho AMPK (Thr172), AMPKα, were obtained from Cell Signaling Technology. Rabbit polyclonal antibody detecting endogenous MCD was obtained from Abcam and antibodies against HADHA and MCAD were from Genetex. A polyclonal antibody for dsRED that reacts with mCherry was obtained from Clontech. Stabilization of Shield-1 was confirmed with a rabbit polyclonal antibody for FKBP-12 (Thermo Scientific). MitoProfile total OXPHOS Rodent WB Antibody cocktail was obtain from MitoSciences. Alpha-Tubulin protein loading control was obtained from Sigma. Gastrocnemius muscle for tissue analysis of signaling proteins, detection of endogenous recombination markers, and stabilization by Shield-1 was harvested and immediately flash frozen in liquid nitrogen. Total protein was extracted by tissue homogenization in cold lysis buffer consisting of 50 mM Tris-HCl, 150 nM NaCl, 1 mM EDTA, 1% Triton X-100, with protease and PhosStop phosphatase inhibitor cocktail (Roche). Tissue homogenates were pelleted at 16,000 *g* for 30 minutes at 4°C and supernatants collected into fresh, cold microcentrifuge tubes. Protein estimation by Pierce BCA Protein Assay Kit was used to determine protein concentration in supernatants. Proteins were separated using NuPAGE Novex 4-12% Bis-Tris Gels in NuPAGE MOPS SDS running buffer or Bio Rad Mini Protean TGX precast gels. Proteins were transferred to PVDF membranes (0.45 μm), blocked in 5% non-fat milk and detected by immunoblotting with the antibodies above. HRP-conjugated secondary antibodies were detected using Amersham ECL Prime Western Blotting Detection Reagent (GE Healthcare) and detected using the FluorChem Western Blot imaging system (Cell Biosciences). Shield-1 was synthesized as previously reported [41,43]. Shield-1 was dried under a stream of nitrogen gas and reconstituted in 50% *N,N*-dimethyacetamide and 50% of a 9:1 PEG-400:Tween-80 mixture [42]. Shield-1 was administered intraperitoneally.

### Animal studies

Animals were housed in a specific pathogen free barrier facility. Tg-fMCD mice were bred to mice expressing Cre from a muscle specific (human alpha-skeletal actin) promoter obtained from Jackson Laboratory to generate Tg-fMCD^skel^ mice [44]. Tg-fMCD^skel^ and control littermates (WT and Cre transgenic) were maintained on a standard chow diet, with free access to food and water and maintained on a 12 hour light-dark photocycle in a temperature controlled environment. At 7 weeks of age, Tg-fMCD^skel^ and control littermate male mice were transitioned from a standard chow diet to a 60% kcal from fat high fat diet (HFD) (D12492, Research Diets, Inc.) or 10% kcal from fat low fat diet (LFD) (D12450J, Research Diets, Inc.). Body weights were measured weekly. Onset of glucose intolerance was assessed by glucose and insulin tolerance tests. At 19 weeks of age, Tg-fMCD^skel^ and control mice were injected i.p. with 60 mg/kg Shield-1 (40 μl formulated in 50% N,N-dimethylacteamide and 50% of a 9:1 PEG-400:Tween-80 mixture) or vehicle alone. Mice received Shield-1 or vehicle injections every 48 hours for 2 weeks. Glucose tolerance tests were repeated on mice to measure efficacy of Shield-1 treatment to lower fasting blood glucose and increase insulin sensitivity. Mice used for the insulin stimulation studies, were i.p. injected with 60 mg/kg Shield-1 or vehicle every 24 hours for 5 days before the stimulation. Acute insulin stimulation was performed on mice following a 6 hour fast during the light cycle. Mice were injected i.p. with 1U/kg insulin (Sigma, bovine pancreas). Tissues were collected 10 minutes after insulin injection, frozen in liquid nitrogen, and stored at -80°C. Animal experiments were done in accordance with the National Institutes of Health Guide for the Care and Use of Laboratory Animals and under the approval of the Johns Hopkins Medical School Animal Care and Use Committee.

### Glucose and insulin tolerance testing

Mice were fasted for 6 hours before i.p. injection with 1.25 mg/g glucose or 0.8 U/kg of insulin (Sigma, bovine pancreas) in a 0.9% NaCl solution. Blood glucose was assayed from tail blood at times, 0, 15 min, 30 min, 60 min, 120 min for the GTT after glucose injection. Blood glucose was assayed from tail blood at times, 0, 15 min, 30 min, 60 min after the insulin injection for the ITT. Serum insulin was collected at the 15 minute time point during the GTT and measured using a mouse insulin ELcISA kit (Millipore).

### Malonyl-CoA decarboxylase assay

Isolated gastrocnemius muscles were assayed for MCD activity following drug and diet treatments via a fluorometric assay as previously described [40,45].

### Quantitative real time PCR analysis

Isolated gastrocnemius muscles from Tg-fMCD^skel^ and control male mice were frozen in liquid nitrogen and stored at -80°C until homogenization with Trizol (Life Technologies) to isolate RNA. The conversion of RNA to cDNA, was performed by using a high capacity cDNA reverse transcription kit (Applied Biosystems). The following PCR primer pairs were used for this study:

*CPT1B* forward, 5′- GGTCCCATAAGAAACAAGACCTCC-3′, *CPTIB* reverse, 5′- CAGAAAGTACCTCAGCCAGGAAAG-3′, *MCAD forward,* 5′-GTTGAACTCGCTAGGCTCAGTTAC-3′, *MCAD reverse,* 5′-CTCTGTGTTGAATCCATAGCCTCC-3′, *PPAR alpha forward, 5′*-ACAAGGCCTCAGGGTACCA-*3′, PPAR alpha reverse, 5′-*GCCGAAAGAAGCCCTTACAG-*3′, PGC1alpha forward, 5′*-CAGCCTCTTTGCCCAGATCT-*3′,* PGC1alpha reverse, 5′-CCGCTAGCAAGTTTGCCTCA-3′, ACOT1 forward, 5′-GACAAGAAGAGCTTCATTCCCGTG-3′, ACOT1 reverse, 5′-CATCAGCATAGAACTCGCTCTTCC-3, 18S rRNA forward, 5′-GCAATTATTCCCCATGAACG-3′, 18 s rRNA reverse, 5′-GGCCTCACTAAACCATCCAA -3.

### Statistics

Statistical analyses were performed using one-way or two-way ANOVA as indicated in the figure legends. Significance is defined when p < 0.05. Data is represented as mean ± SEM.

## Results

### In Vivo chemical-genetic regulation of Malonyl-CoA decarboxylase in skeletal muscle

**Figure 1:**
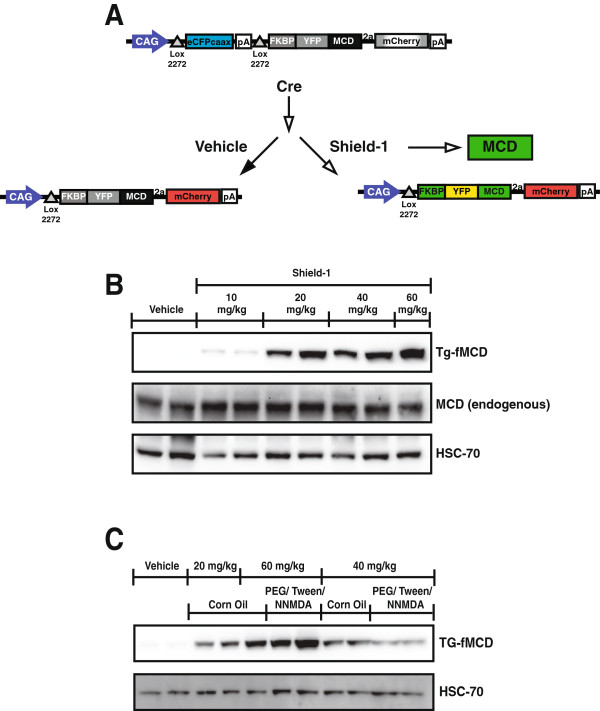
**Tissue specific chemically inducible Malonyl-CoA Decarboxylase. (A)** Schematic diagram of the dually regulated MCD transgene. **(B)** Tg-fMCD^skel^ mice were injected i.p. with vehicle or Shield-1 at various doses. Samples were collected 24 hours after injection and gastrocnemius muscle samples were probed for the indicated proteins. **(C)** Dose and delivery vehicle analysis of Shield-1 in Tg-fMCD^skel^ mice. Gastrocnemius muscles from mice injected i.p. with vehicle or Shield-1 at varying doses and delivery methods were collected 24 hours post treatment to determine efficacy of Shield-1 stabilization by western blot for FKBP12.

Lipids mediate insulin resistance in skeletal muscle via an ill-defined mechanism; however, promoting the rate of fatty acid oxidation in skeletal muscle has been proposed to affect insulin sensitivity in this tissue [30,32,46-50]. Given the importance of MCD in regulating skeletal muscle fatty acid oxidation, we generated transgenic mice where MCD (Tg-fMCD) can be regulated in a cell and chemical specific manner in order to determine the effect of acutely altering fatty acid metabolism in insulin resistance [40]. A cytoplasmic targeted MCD fused to a destabilization domain was cloned downstream of a lox mCherry stop cassette. Therefore, the expression of the transgene is controlled in a Cre-recombinase dependent manner. The destabilization domain was derivatized from FKBP12 (FK506 binding protein 12) enabling reversible and dose dependent protein stabilization with its synthetic ligand, Shield-1 (fMCD) [41]. In order to target the transgene to skeletal muscle, Tg-fMCD mice were bred to mice expressing Cre recombinase from the human alpha skeletal muscle actin promoter, ACTA1, producing Tg-fMCD^Skel^ mice. Thus, we generated mice expressing cytoplasmic MCD that is stabilized by Shield-1 in a skeletal muscle specific manner (Figure 1A).

In order to determine the appropriate dose to achieve effective transgene stabilization we injected Tg-fMCD^Skel^ mice with increasing concentrations of Shield-1 in corn oil to determine the required dose to effectively increase MCD. Shield-1 induced effective transgene stabilization at 20 mg/kg and in a dose-dependent manner with the highest degree of stabilization at the 60 mg/kg dose (Figure 1B) [40]. However, since our goal was to determine the effect of lipid metabolism on insulin sensitivity, we chose a vehicle other than corn oil to prevent confounding effects of the lipid. Others have reported *in vivo* delivery of Shield-1 in PEG/Tween/NNMDA [42]. Injection of 60 mg/kg Shield-1 with a PEG/Tween/NNMDA vehicle most effectively stabilized the MCD transgene (Figure 1C). Therefore we chose 60 mg/kg Shield-1 in a PEG/Tween/NNMDA vehicle to alter MCD in skeletal muscle in mice.

### An acute induction of MCD in skeletal muscle did not alter body weight or glucose sensitivity

**Figure 2:**
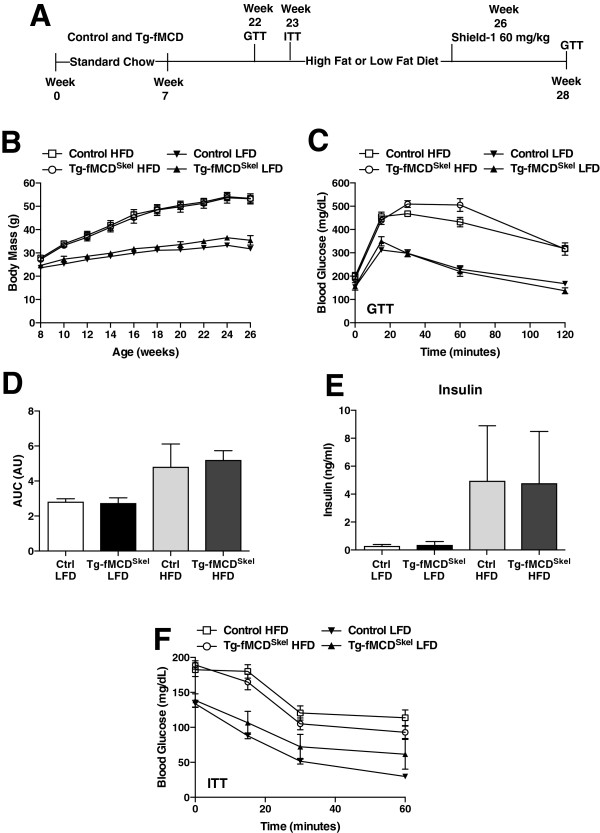
**The development of diet-induced obesity and insulin resistance in Tg-fMCD**^**skel **^**mice. (A)** Experimental timeline of control and Tg-fMCD^skel^ mice placed on low fat diet (LFD) or high fat diet (HFD) followed by treatment of Shield-1 to induce MCD within the skeletal muscle. **(B)** Mice were weighed weekly to monitor body weight gain. **(C)** Confirmation of glucose intolerance by glucose tolerance test (GTT). **(D)** Area under the curve for GTT and **(E)** insulin at 15 min after glucose challenge in control and Tg-fMCD^skel^ mice. **(F)** Insulin tolerance test (ITT) confirms insulin resistance in mice fed the high fat diet. (n = 5-8) Data are expressed as means +/- SEM.

To assess whether an acute increase in fatty acid oxidation is associated with improvements in body weight and insulin sensitivity in a model of diet induced obesity, we made Tg-fMCD^Skel^ and littermate controls obese and insulin resistant with 15 weeks of high fat diet (HFD) (60% kcal from fat) feeding. An additional group of Tg-fMCD^Skel^ and littermate controls were fed a low fat diet (LFD) (10% kcal from fat) for 15 weeks (Figure 2A). Tg-fMCD^Skel^ and control mice on the high fat diet rapidly gained weight, and there was no difference between genotypes (Figure 2B). Mice on the LFD remained lean (Figure 2B). Glucose sensitivity was examined by performing glucose and insulin tolerance tests. High fat diet-induced glucose intolerance was clearly evident in both genotypes (Figure 2C-E). Tg-fMCD^Skel^ and control mice began with elevated fasting blood glucose levels in the insulin tolerance test and failed to clear blood glucose as efficiently as their lean counterparts on the LFD (Figure 2F). Thus, as expected, Tg-fMCD^Skel^ mice in the absence of the inducing ligand displayed no protection from obesity or insulin resistance.

**Figure 3:**
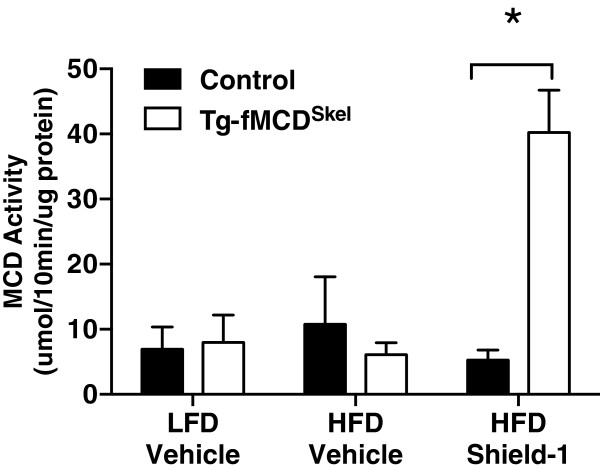
**Chemical-genetic induction of MCD activity in skeletal muscle.** Malonyl-CoA decarboxylase activity in Tg-fMCD^skel^ and control mice fed low fat and high fat diets treated with Shield-1 (60 mg/kg) or vehicle. (n = 3-4) Data are expressed as means +/- SEM. *p < 0.001 by 2-way ANOVA.

**Figure 4:**
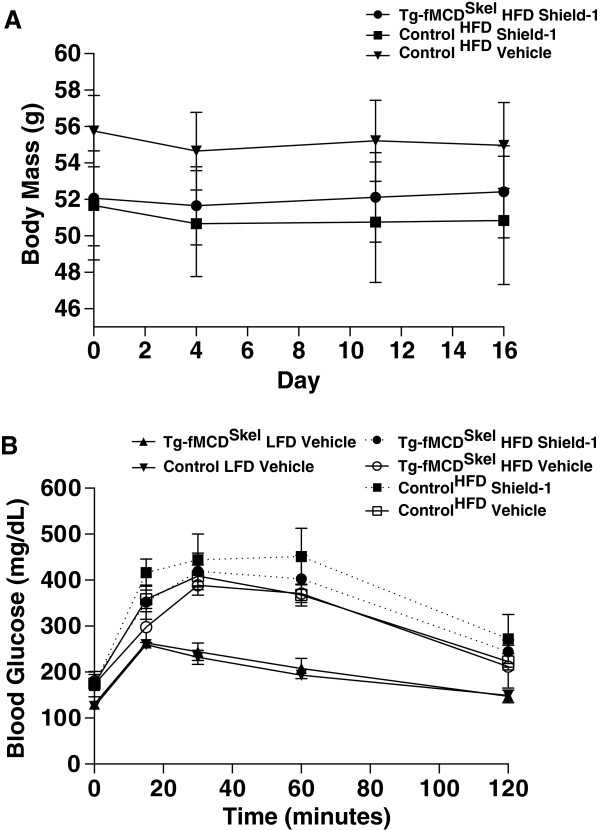
**Acute induction of MCD does not reverse diet induced obesity or glucose intolerance**. Tg-fMCD^skel^ and control mice on HFD and LFD received 8 doses of Shield-1 (60 mg/kg) or vehicle control over two weeks. **(A)** Body weights were measured before and after Shield-1 treatment. **(B)** Mice were subjected to a GTT to determine glucose tolerance. (n = 3-10) Data are expressed as means +/- SEM.

To gain insight into the role of MCD in regulating metabolic dysfunction in the skeletal muscle, we treated Tg-fMCD^Skel^ and control HFD-induced obese and insulin resistant mice with Shield-1 (60 mg/kg) or vehicle every 48 hours for 2 weeks. This dose of shield increased gastrocnemius MCD activity greater than 5-fold (Figure 3). Despite this large increase in skeletal muscle MCD activity, Shield-1 treatment did not alter body weight in Tg-fMCD^Skel^ or control HFD mice (Figure 4A). Moreover, MCD induction by Shield-1 did not alter glucose sensitivity of these mice (Figure 4B). Insulin measurements from Tg-fMCD^Skel^ HFD Shield-1 treated mice taken during the glucose tolerance test, show no difference in insulin sensitivity compared to control HFD Shield-1 controls; both groups of mice remained insulin resistant compared to lean controls. These data suggest that acute induction of MCD in the skeletal muscle is not sufficient to alter adiposity or insulin sensitivity.

### Acute induction of MCD in skeletal muscle repressed insulin signaling

**Figure 5:**
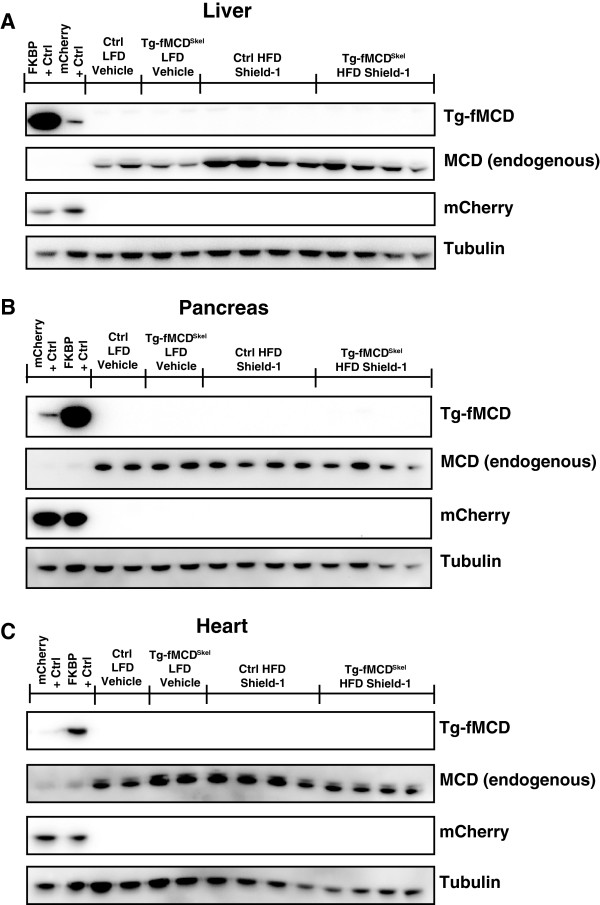
**MCD transgene expression and Shield-1 stabilization is specific to skeletal muscle.** Western blot of **(A)** liver **(B)** pancreas and **(C)** heart tissue extracted from Tg-fMCD^skel^ and control mice on HFD and LFD treated with Shield-1 (5 doses at 60 mg/kg) or vehicle. Samples were blotted for FKBP12 (for transgene stabilization), MCD (endogenous expression), mCherry (transgene expression), and alpha Tubulin (loading control). Positive controls for mCherry and FKBP are derived from skeletal muscle of Tg-fMCD^skel^ mice treated with vehicle or Shield-1.

To address the specificity of the MCD transgene for recombination and induction, we collected the liver, pancreas and heart which highly express the non-recombined transgene [40]. None of these tissues expressed mCherry (a marker for recombination) or expressed the Tg-fMCD (Shield-1 stabilized MCD) transgene (Figure 5).

**Figure 6:**
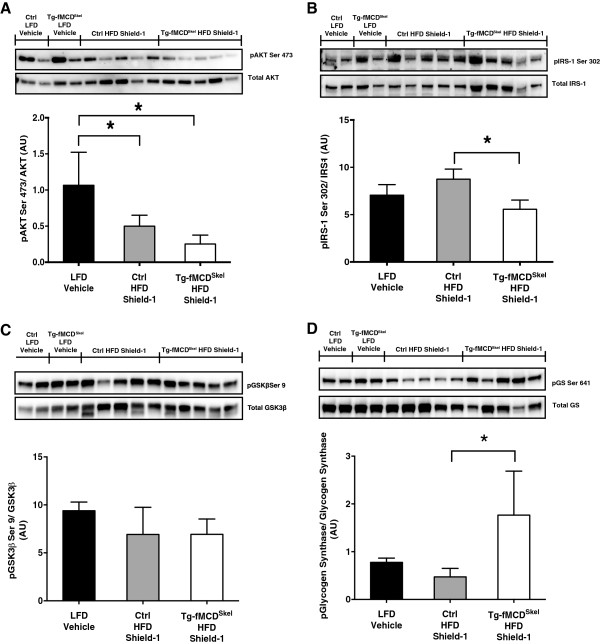
**MCD induction exacerbates defects in diet-induced insulin signaling.** Western blot of gastrocnemius muscle isolated from Tg-fMCD^skel^ and control mice on LFD or HFD given Shield-1 (60 mg/kg) or vehicle for 5 days. Mice were fasted for 6 hours before a 10 min insulin stimulation. **(A)** AKT Ser 473 phosphorylation, normalized to total protein and quantified. **(B)** IRS-1 Ser 302 phosphorylation, normalized to total IRS-1 and quantified. **(C)** GSK3β Ser 9 phosphorylation, normalized to total GSK3β and quantified. **(D)** Glycogen Synthase (GS) Ser 641 phosphorylation, normalized to total Glycogen Synthase and quantified. Data are expressed as means +/- SEM. *p < 0.05 by one-way ANOVA.

To address possible tissue-specific insulin sensitivity in the skeletal muscle, we performed an *in vivo* insulin stimulation followed by tissue collection. HFD fed Tg-fMCD^Skel^ and control mice received an acute treatment of Shield-1 at 60 mg/kg every 24 hours for 5 days. Six hour fasted mice were injected with insulin 24 hours after the last dose of Shield-1 and gastrocnemius muscle was harvested 10 minutes after the insulin injection. As expected insulin stimulated phosphorylation of AKT Ser^473^ was decreased in Tg-fMCD^Skel^ HFD and control HFD mice compared to lean LFD controls (Figure 6A). Tg-fMCD^Skel^ HFD Shield-1 mice, compared to control HFD Shield-1 controls, showed a further 2-fold suppression in insulin stimulated phosphorylated AKT Ser^473^ relative to total AKT in the skeletal muscle (Figure 6A). Additionally, IRS-1 Ser^302^ phosphorylation was lower in Tg-fMCD^Skel^ HFD gastrocnemius compared to all groups (Figure 6B). Although, GSK3β Ser^9^ phosphorylation was not different between groups (Figure 6C), glycogen synthase Ser^641^ phosphorylation was enhanced in Tg-fMCD^Skel^ HFD gastrocnemius (Figure 6D). Taken together these data suggest that induction of MCD in skeletal muscle exacerbates HFD-induced insulin resistance evidenced by decreased insulin signaling.

### Induction of MCD suppresses genes of fatty acid oxidation

**Figure 7:**
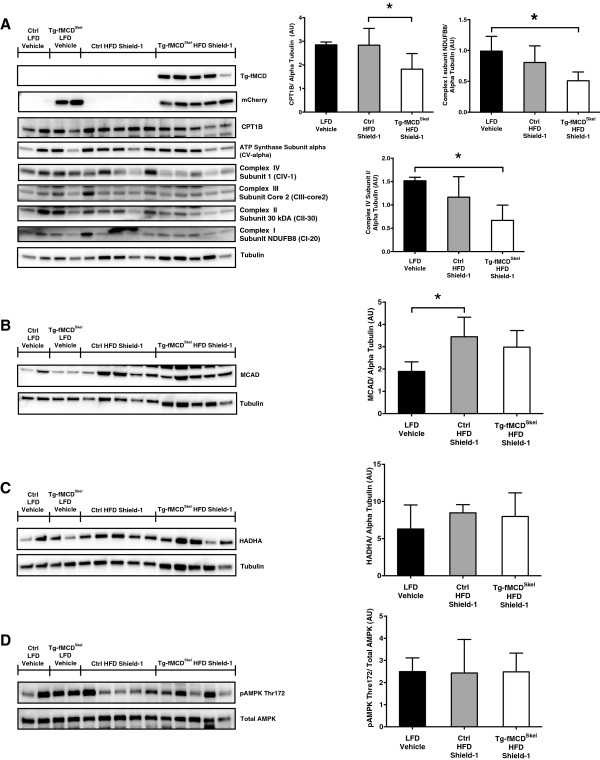
**MCD induction suppresses the fatty acid oxidative pathway.** Western blot of gastrocnemius muscle for isolated from Tg-fMCD^skel^ and control mice on LFD or HFD given Shield-1 (60 mg/kg) or vehicle for 5 days. **(A)** CPT1B and OXPHOS complexes (as indicated) were normalized to alpha Tubulin and quantified. Samples were blotted with FKBP12 (for transgene stabilization), mCherry (transgene expression) and alpha Tubulin (loading control). **(B)** MCAD and **(C)** HADHA were normalized to alpha Tubulin and quantified **(D)** AMPK Thr 172 phophorylation was determined, normalized for total AMPK and quantified. Data are expressed as means +/- SEM. *p < 0.05 by one-way ANOVA.

Previously, we showed that an acute induction of MCD leads to an increase in fatty acid oxidation *in vivo*[40]. Because malonyl-CoA and MCD are major regulators of skeletal muscle fatty acid oxidation, we examined the effect of inducing MCD on genes in the fatty acid oxidation pathway in obese and insulin resistant mice. Surprisingly, Tg-fMCD^Skel^ HFD Shield-1 treated mice, compared to control HFD Shield-1 treated counterparts, had approximately a 2-fold reduced protein abundance of CPT1B and oxidative phosphorylation complex proteins (Figure 7A). Additionally, we saw Medium Chain Acyl-CoA Dehydrogenase (MCAD) increase in high fat fed mice without an effect of MCD induction (Figure 7B) while Hydroxyacyl-CoA Dehydrogenase alpha subunit (HADHA) did not change between groups (Figure 7C). Due to reduced fatty acid oxidation protein expression, we questioned the possible activation of the energy sensor, AMPK. AMPK phosphorylation at Thr172, the major activating phosphorylation site was not changed by MCD induction (Figure 7D). These data suggest that alterations in AMPK do not play a role in mediating the decrease in insulin sensitivity observed in Tg-fMCD^Skel^ mice.

**Figure 8:**
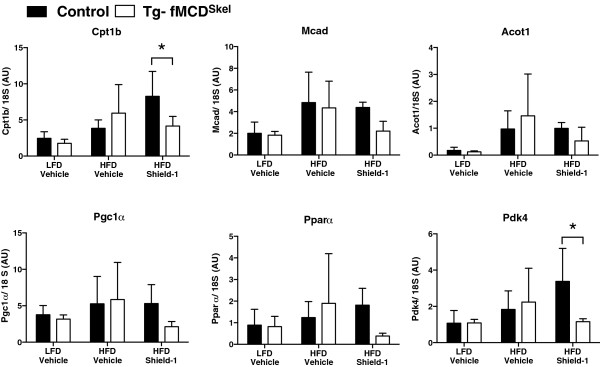
**Fatty acid oxidation genes are suppressed upon induction of MCD.** qRT-PCR analysis of Cpt1b, Mcad, Acot1, Pgc1α, Pparα and Pdk4 in gastrocnemius muscle from Tg-fMCD^skel^ and control mice on LFD or HFD given Shield-1 (60 mg/kg) or vehicle for 5 days. Data are expressed as means +/- SEM. *p < 0.05 by 2-way ANOVA.

Because we observed a decrease in CPT1B and components of oxidative phosphorylation, we determined if the decrease was due to regulation at the transcriptional level. Gene expression analysis of gastrocnemius muscle from Tg-fMCD^Skel^ HFD Shield-1 treated mice demonstrated a decrease in Cpt1b mRNA upon MCD induction (Figure 8). The transcriptional regulatory genes Pparα and Pgc1α were unchanged after MCD induction as well as the canonical Pparα target Acot1 (Figure 8). Owning to a possible compensation by carbohydrate oxidation, Pyruvate Dehydrogenase Kinase 4 (Pdk4) was transcriptionally suppressed (Figure 8). These data demonstrate that an acute change in the regulation of skeletal muscle lipid oxidation by MCD in a model of obesity induces a concomitant reduction in the protein and mRNA abundance in genes of fatty acid oxidation.

## Discussion

The role of skeletal muscle fatty oxidation in obesity and glucose intolerance is not clear. A growing number of studies have shown a detrimental effect of skeletal muscle mitochondrial fatty acid oxidation in diet induced insulin resistance and obesity [5]. We have increased MCD to increase the potential for fatty acid oxidation in skeletal muscle of obese mice. Here, we demonstrate that 1) induction of MCD did not lead to changes in body weight in HFD induced obese mice, 2) an acute induction of MCD augmented defects in skeletal muscle insulin signaling in HFD induced obese mice, 3) MCD induction resulted in a suppression of oxidative genes within skeletal muscle. The induction of MCD in the skeletal muscle exacerbated the diabetic phenotype by negatively affecting insulin signaling. These data provide insight into the pathophysiology of skeletal muscle insulin resistance and shows that inducing MCD in already pathogenic mice to facilitate increased fatty acid oxidation does not reverse obesity or glucose intolerance.

The role of skeletal muscle mitochondria in the promotion or protection from metabolic dysfunction is not well understood. Based on the strong correlation between increased lipid consumption and insulin resistance, some have suggested that the accumulation of cytoplasmic lipid intermediates that are often seen in diabetic patients and animal models directly impairs insulin signaling [19,51]. Based on this, and the observation that a sedentary lifestyle promotes metabolic dysfunction, it has been suggested that the stimulation of fatty acid oxidation could lower the concentration of lipotoxic intermediates to improve insulin sensitivity by removing the lipid substrates. In support of this, individuals with type 2 diabetes and even pre-diabetes have decreased mitochondrial function [13,18,52,53]. However, several mouse models with increased lipid oxidation in skeletal muscle do not have improved insulin sensitivity [25]. Inversely, metformin, which is widely used to treat diabetic patients, has been proposed to work as a mild electron transport inhibitor [54-56]. Also, mice with suppressed function of complex I of the electron transport chain in skeletal muscle are protected from diet induced glucose intolerance [57]. In support of these findings, we were unable to observe improvements in body weight or insulin sensitivity by increasing fatty acid oxidative potential with the over-expression of MCD in our model of high fat diet induced insulin resistance.

Obesity induced insulin resistance is associated with alterations in fatty oxidation genes and mitochondrial dysfunction [58,59]. Human studies support the observation of decreased transcriptional control of fatty acid oxidative genes in the skeletal muscle of obese, highly insulin resistant people. Specifically, individuals in the most insulin resistant and insulin sensitive groups had lowered expression of Pgc1α, Pparα, and Cpt1b [60]. A second study described decreased mRNA content in Pdk4, Pgc1α, and Pparα in obese individuals [61]. In contrast, decreasing fatty acid oxidation by the small molecule inhibition of CPT1, improved insulin sensitivity and increased pyruvate dehydrogenase activity to promote glucose oxidation, and AKT phosphorylation in mice [62]. These studies suggest the skeletal muscle employs different mechanisms to adapt to varying degrees of insulin resistance. The skeletal muscle may use alternate mechanisms to regulate macronutrient substrate switching to increase glucose oxidation in a prolonged state of over nutrition. [33,63].

Skeletal muscle MCD regulates the concentration of malonyl-CoA, the precursor for fatty acid synthesis and elongation. Decreasing the concentration of malonyl-CoA, dysinhibits CPT1, the rate-setting enzyme in mitochondrial fatty acid oxidation. Here we showed that the induction of MCD and thereby increased fatty acid oxidation potential in skeletal muscle in the absence of increased ATP utilization or uncoupling may be a contributing factor in diabetes. That is, unbalancing the flux of macronutrient metabolism from mitochondrial capacity may be an underlying cause of metabolic dysfunction. Interestingly, acute MCD expression in the skeletal muscle down-regulated Cpt1b and other genes in the lipid oxidation pathway at the transcriptional and protein level. In support of this data, MCD knockout mouse hearts or canine hearts subjected to pharmacologic inhibition of fatty acid oxidation, showed the inverse transcriptional alterations [64,65]. These fatty acid oxidation genes are known targets of Pparα transcriptional activation, suggesting a novel mechanism linking intermediary metabolism to Pparα transcriptional regulation. Pparα has been shown to play an important role in the transcriptional regulation of lipid and glucose metabolism, particularly in skeletal muscle fatty acid oxidation [66]. Studies have suggested a wide variety of lipids that function as endogenous Ppar activators [67,68]. Metabolites likely play larger roles in regulating genes and pathways than has been appreciated. We suggest a possible mechanism where increasing MCD results in increased fatty acid intermediates to generate metabolic signals that affect Pparα mediated transcriptional control.

## Conclusions

Induction of MCD in pathogenic obese and glucose intolerant skeletal muscle does not improve obesity or insulin resistance. Induction of MCD leads to decreased fatty acid oxidation gene expression, and impaired skeletal muscle insulin signaling. These results suggest that increasing mitochondrial fatty acid oxidative flux in the absence of energy demand contributes to lipid induced insulin resistance.

## Abbreviations

ACC2: Acetyl CoA Carboxylase-2; PDK4: Pyruvate dehydrogenase kinase, isozyme 4; AMPK: 5′ adenosine monophosphate activated protein Kinase; CoA: Coenzyme A; CPT1B: Carnitine palmitoyltransferase 1B; FKBP12: FK506 binding protein 12; KO: Knockout; MCD: Malonyl-CoA decarboxylase; MCAD: Medium chain Acyl-CoA dehydrogenase; HADHA: Hydroxyacyl-CoA dehydrogenase alpha; PGC1α: Peroxisome proliferator-activated receptor gamma coactivator 1 alpha; PPAR: Peroxisome proliferator-activated receptor; WT: Wild-type; Ctrl: Control.

## Competing interests

The authors declare that they no competing interests.

## Authors’ contributions

MJW conceived of the project, collected samples and aided in writing. SR interpreted results and wrote the manuscript. SR and JME collected samples and performed experiments. All authors read and approved the final manuscript.

## References

[B1] DeFronzo RA Gunnarsson R Bjorkman O Olsson M Wahren J Effects of insulin on peripheral and splanchnic glucose metabolism in noninsulin-dependent (type II) diabetes mellitus J Clin Invest 1985 76 1 149 155 3894418 10.1172/JCI111938 PMC423730

[B2] Warram JH Martin BC Krolewski AS Soeldner JS Kahn CR Slow glucose removal rate and hyperinsulinemia precede the development of type II diabetes in the offspring of diabetic parents Ann Intern Med 1990 113 12 909 915 2240915 10.7326/0003-4819-113-12-909

[B3] Kelley DE Williams KV Price JC McKolanis TM Goodpaster BH Thaete FL Plasma fatty acids, adiposity, and variance of skeletal muscle insulin resistance in type 2 diabetes mellitus J Clin Endocrinol Metabol 2001 86 11 5412 5419 10.1210/jcem.86.11.8027 11701715

[B4] Lumeng CN Saltiel AR Inflammatory links between obesity and metabolic disease J Clin Invest 2011 121 6 2111 2117 21633179 10.1172/JCI57132 PMC3104776

[B5] Muoio DM Neufer PD Lipid-induced mitochondrial stress and insulin action in muscle Cell Metab 2012 15 5 595 605 22560212 10.1016/j.cmet.2012.04.010 PMC3348508

[B6] Morino K Petersen KF Shulman GI Molecular mechanisms of insulin resistance in humans and their potential links with Mitochondrial Dysfunction Diabetes 2006 55 Supplement_2 S9 S15 17130651 10.2337/db06-S002 PMC2995546

[B7] Muoio DM Newgard CB Mechanisms of disease: molecular and metabolic mechanisms of insulin resistance and beta-cell failure in type 2 diabetes Nat Rev Mol Cell Biol 2008 9 3 193 205 18200017 10.1038/nrm2327

[B8] Holland WL Brozinick JT Wang LP Hawkins ED Sargent KM Liu Y Narra K Hoehn KL Knotts TA Siesky A Nelson DH Karathanasis SK Fontenot GK Birnbaum MJ Summers SA Inhibition of ceramide synthesis ameliorates glucocorticoid-, saturated-fat-, and obesity-induced insulin resistance Cell Metab 2007 5 3 167 179 17339025 10.1016/j.cmet.2007.01.002

[B9] Pan DA Lillioja S Kriketos AD Milner MR Baur LA Bogardus C Jenkins AB Storlien LH Skeletal muscle triglyceride levels are inversely related to insulin action Diabetes 1997 46 6 983 988 9166669 10.2337/diab.46.6.983

[B10] Kim JK Fillmore JJ Sunshine MJ Albrecht B Higashimori T Kim DW Liu ZX Soos TJ Cline GW O’Brien WR Littman DR Shulman GI PKC-theta knockout mice are protected from fat-induced insulin resistance J Clin Invest 2004 114 6 823 827 15372106 10.1172/JCI22230 PMC516267

[B11] Stratford S DeWald DB Summers SA Ceramide dissociates 3′-phosphoinositide production from pleckstrin homology domain translocation Biochem J 2001 354 Pt 2 359 368 11171115 10.1042/0264-6021:3540359 PMC1221664

[B12] Summers SA Garza LA Zhou H Birnbaum MJ Regulation of insulin-stimulated glucose transporter GLUT4 translocation and Akt kinase activity by ceramide Mol Cell Biol 1998 18 9 5457 5464 9710629 10.1128/mcb.18.9.5457 PMC109130

[B13] Kelley DE He J Menshikova EV Ritov VB Dysfunction of mitochondria in human skeletal muscle in type 2 diabetes Diabetes 2002 51 10 2944 2950 12351431 10.2337/diabetes.51.10.2944

[B14] He J Watkins S Kelley DE Skeletal muscle lipid content and oxidative enzyme activity in relation to muscle fiber type in type 2 diabetes and obesity Diabetes 2001 50 4 817 823 11289047 10.2337/diabetes.50.4.817

[B15] Petersen KF Befroy D Dufour S Dziura J Ariyan C Rothman DL DiPietro L Cline GW Shulman GI Mitochondrial dysfunction in the elderly: possible role in insulin resistance Science 2003 300 5622 1140 1142 12750520 10.1126/science.1082889 PMC3004429

[B16] Befroy DE Petersen KF Dufour S Mason GF de Graaf RA Rothman DL Shulman GI Impaired mitochondrial substrate oxidation in muscle of insulin-resistant offspring of type 2 diabetic patients Diabetes 2007 56 5 1376 1381 17287462 10.2337/db06-0783 PMC2995532

[B17] Noland RC Hickner RC Jimenez-Linan M Vidal-Puig A Zheng D Dohm GL Cortright RN Acute endurance exercise increases skeletal muscle uncoupling protein-3 gene expression in untrained but not trained humans Metabol Clin Exp 2003 52 2 152 158 10.1053/meta.2003.50021 12601624

[B18] Hulver MW Berggren JR Cortright RN Dudek RW Thompson RP Pories WJ MacDonald KG Cline GW Shulman GI Dohm GL Houmard JA Skeletal muscle lipid metabolism with obesity Am J Physiol Endocrinol Metab 2003 284 4 E741 E747 12626325 10.1152/ajpendo.00514.2002

[B19] Kim J-Y Hickner RC Cortright RL Dohm GL Houmard JA Lipid oxidation is reduced in obese human skeletal muscle Am J Physiol Endocrinol Metabol 2000 279 5 E1039 E1044 10.1152/ajpendo.2000.279.5.E1039 11052958

[B20] Krssak M Falk Petersen K Dresner A DiPietro L Vogel SM Rothman DL Roden M Shulman GI Intramyocellular lipid concentrations are correlated with insulin sensitivity in humans: a 1H NMR spectroscopy study Diabetologia 1999 42 1 113 116 10027589 10.1007/s001250051123

[B21] Goodpaster BH Theriault R Watkins SC Kelley DE Intramuscular lipid content is increased in obesity and decreased by weight loss Metabol Clin Exp 2000 49 4 467 472 10.1016/s0026-0495(00)80010-4 10778870

[B22] Sacksteder KA Morrell JC Wanders RJ Matalon R Gould SJ MCD encodes peroxisomal and cytoplasmic forms of malonyl-CoA decarboxylase and is mutated in malonyl-CoA decarboxylase deficiency J Biol Chem 1999 274 35 24461 24468 10455107 10.1074/jbc.274.35.24461

[B23] Pender C Trentadue AR Pories WJ Dohm GL Houmard JA Youngren JF Expression of genes regulating malonyl-CoA in human skeletal muscle J Cell Biochem 2006 99 3 860 867 16721829 10.1002/jcb.20944

[B24] Funai K Song H Yin L Lodhi IJ Wei X Yoshino J Coleman T Semenkovich CF Muscle lipogenesis balances insulin sensitivity and strength through calcium signaling J Clin Invest 2013 123 3 1229 1240 23376793 10.1172/JCI65726 PMC3582136

[B25] Hoehn KL Turner N Swarbrick MM Wilks D Preston E Phua Y Joshi H Furler SM Larance M Hegarty BD Leslie SJ Pickford R Hoy AJ Kraegen EW James DE Cooney GJ Acute or chronic upregulation of mitochondrial fatty acid oxidation has no net effect on whole-body energy expenditure or adiposity Cell Metab 2010 11 1 70 76 20074529 10.1016/j.cmet.2009.11.008 PMC2824926

[B26] Olson DP Pulinilkunnil T Cline GW Shulman GI Lowell BB Gene knockout of Acc2 has little effect on body weight, fat mass, or food intake Proc Natl Acad Sci U S A 2010 107 16 7598 7603 20368432 10.1073/pnas.0913492107 PMC2867727

[B27] Rasmussen BB Holmback UC Volpi E Morio-Liondore B Paddon-Jones D Wolfe RR Malonyl coenzyme A and the regulation of functional carnitine palmitoyltransferase-1 activity and fat oxidation in human skeletal muscle J Clin Invest 2002 110 11 1687 1693 12464674 10.1172/JCI15715 PMC151631

[B28] Bouzakri K Austin R Rune A Lassman ME Garcia-Roves PM Berger JP Krook A Chibalin AV Zhang BB Zierath JR Malonyl CoenzymeA decarboxylase regulates lipid and glucose metabolism in human skeletal muscle Diabetes 2008 57 6 1508 1516 18314420 10.2337/db07-0583

[B29] Ussher JR Koves TR Jaswal JS Zhang L Ilkayeva O Dyck JR Muoio DM Lopaschuk GD Insulin-stimulated cardiac glucose oxidation is increased in high-fat diet-induced obese mice lacking malonyl CoA decarboxylase Diabetes 2009 58 8 1766 1775 19478144 10.2337/db09-0011 PMC2712785

[B30] Abu-Elheiga L Matzuk MM Abo-Hashema KA Wakil SJ Continuous fatty acid oxidation and reduced fat storage in mice lacking acetyl-CoA carboxylase 2 Science 2001 291 5513 2613 2616 11283375 10.1126/science.1056843

[B31] An J Muoio DM Shiota M Fujimoto Y Cline GW Shulman GI Koves TR Stevens R Millington D Newgard CB Hepatic expression of malonyl-CoA decarboxylase reverses muscle, liver and whole-animal insulin resistance Nat Med 2004 10 3 268 274 14770177 10.1038/nm995

[B32] Choi CS Savage DB Abu-Elheiga L Liu Z-X Kim S Kulkarni A Distefano A Hwang Y-J Reznick RM Codella R Zhang D Cline GW Wakil SJ Shulman GI Continuous fat oxidation in acetyl–CoA carboxylase 2 knockout mice increases total energy expenditure, reduces fat mass, and improves insulin sensitivity Proc Natl Acad Sci 2007 104 42 16480 16485 17923673 10.1073/pnas.0706794104 PMC2034222

[B33] Koves TR Ussher JR Noland RC Slentz D Mosedale M Ilkayeva O Bain J Stevens R Dyck JR Newgard CB Lopaschuk GD Muoio DM Mitochondrial overload and incomplete fatty acid oxidation contribute to skeletal muscle insulin resistance Cell Metab 2008 7 1 45 56 18177724 10.1016/j.cmet.2007.10.013

[B34] Brown GK Scholem RD Bankier A Danks DM Malonyl coenzyme A decarboxylase deficiency J Inherit Metab Dis 1984 7 1 21 26 6145813 10.1007/BF01805615

[B35] Matalon R Michaels K Kaul R Whitman V Rodriguez-Novo J Goodman S Thorburn D Malonic aciduria and cardiomyopathy J Inherit Metab Dis 1993 16 3 571 573 7609455 10.1007/BF00711684

[B36] Yano S Sweetman L Thorburn DR Mofidi S Williams JC A new case of malonyl coenzyme A decarboxylase deficiency presenting with cardiomyopathy Eur J Pediatr 1997 156 5 382 383 9177981 10.1007/s004310050619

[B37] de Wit MCY de Coo IFM Verbeek E Schot R Schoonderwoerd GC Duran M de Klerk JBC Huijmans JGM Lequin MH Verheijen FW Mancini GMS Brain abnormalities in a case of malonyl-CoA decarboxylase deficiency Mol Genet Metab 2006 87 2 102 106 16275149 10.1016/j.ymgme.2005.09.009

[B38] Salomons GS Jakobs C Pope LL Errami A Potter M Nowaczyk M Olpin S Manning N Raiman JAJ Slade T Champion MP Peck D Gavrilov D Hillman R Hoganson GE Donaldson K Shield JPH Ketteridge D Wasserstein M Gibson KM Clinical, enzymatic and molecular characterization of nine new patients with malonyl-coenzyme A decarboxylase deficiency J Inherit Metab Dis 2007 30 1 23 28 17186413 10.1007/s10545-006-0514-6

[B39] Kerner J Hoppel CL Radiochemical malonyl-CoA decarboxylase assay: activity and subcellular distribution in heart and skeletal muscle Anal Biochem 2002 306 2 283 289 12123667 10.1006/abio.2002.5696

[B40] Rodriguez S Wolfgang MJ Targeted chemical-genetic regulation of protein stability in vivo Chem Biol 2012 19 3 391 398 22444594 10.1016/j.chembiol.2011.12.022 PMC3314227

[B41] Banaszynski LA Chen LC Maynard-Smith LA Ooi AG Wandless TJ A rapid, reversible, and tunable method to regulate protein function in living cells using synthetic small molecules Cell 2006 126 5 995 1004 16959577 10.1016/j.cell.2006.07.025 PMC3290523

[B42] Banaszynski LA Sellmyer MA Contag CH Wandless TJ Thorne SH Chemical control of protein stability and function in living mice Nat Med 2008 14 10 1123 1127 18836461 10.1038/nm.1754 PMC2605277

[B43] Yang W Rozamus LW Narula S Rollins CT Yuan R Andrade LJ Ram MK Phillips TB van Schravendijk MR Dalgarno D Clackson T Holt DA Investigating protein-ligand interactions with a mutant FKBP possessing a designed specificity pocket J Med Chem 2000 43 6 1135 1142 10737745 10.1021/jm9904396

[B44] Miniou P Tiziano D Frugier T Roblot N Le Meur M Melki J Gene targeting restricted to mouse striated muscle lineage Nucleic Acids Res 1999 27 19 e27 e30 10481039 10.1093/nar/27.19.e27 PMC148637

[B45] Saha AK Schwarsin AJ Roduit R Masse F Kaushik V Tornheim K Prentki M Ruderman NB Activation of malonyl-CoA decarboxylase in rat skeletal muscle by contraction and the AMP-activated protein kinase activator 5-aminoimidazole-4-carboxamide-1-beta -D-ribofuranoside J Biol Chem 2000 275 32 24279 24283 10854420 10.1074/jbc.C000291200

[B46] Bruce CR Hoy AJ Turner N Watt MJ Allen TL Carpenter K Cooney GJ Febbraio MA Kraegen EW Overexpression of Carnitine Palmitoyltransferase-1 in skeletal muscle is sufficient to enhance fatty acid oxidation and improve high-fat diet–induced insulin resistance Diabetes 2009 58 3 550 558 19073774 10.2337/db08-1078 PMC2646053

[B47] Abu-Elheiga L Oh W Kordari P Wakil SJ Acetyl-CoA carboxylase 2 mutant mice are protected against obesity and diabetes induced by high-fat/high-carbohydrate diets Proc Natl Acad Sci 2003 100 18 10207 10212 12920182 10.1073/pnas.1733877100 PMC193540

[B48] Henique C Mansouri A Fumey G Lenoir V Girard J Bouillaud F Prip-Buus C Cohen I Increased mitochondrial fatty acid oxidation is sufficient to protect skeletal muscle cells from palmitate-induced apoptosis J Biol Chem 2010 285 47 36818 36827 20837491 10.1074/jbc.M110.170431 PMC2978610

[B49] Perdomo G Commerford SR Richard A-MT Adams SH Corkey BE O’Doherty RM Brown NF Increased β-oxidation in muscle cells enhances insulin-stimulated glucose metabolism and protects against fatty acid-induced insulin resistance despite intramyocellular lipid accumulation J Biol Chem 2004 279 26 27177 27186 15105415 10.1074/jbc.M403566200

[B50] Power RA Hulver MW Zhang JY Dubois J Marchand RM Ilkayeva O Muoio DM Mynatt RL Carnitine revisited: potential use as adjunctive treatment in diabetes Diabetologia 2007 50 4 824 832 17310372 10.1007/s00125-007-0605-4 PMC5682624

[B51] Kim JK Fillmore JJ Chen Y Yu C Moore IK Pypaert M Lutz EP Kako Y Velez-Carrasco W Goldberg IJ Breslow JL Shulman GI Tissue-specific overexpression of lipoprotein lipase causes tissue-specific insulin resistance Proc Natl Acad Sci U S A 2001 98 13 7522 7527 11390966 10.1073/pnas.121164498 PMC34701

[B52] Ukropcova B McNeil M Sereda O de Jonge L Xie H Bray GA Smith SR Dynamic changes in fat oxidation in human primary myocytes mirror metabolic characteristics of the donor J Clin Invest 2005 115 7 1934 1941 16007256 10.1172/JCI24332 PMC1159139

[B53] Weiss R Dufour S Taksali SE Tamborlane WV Petersen KF Bonadonna RC Boselli L Barbetta G Allen K Rife F Savoye M Dziura J Sherwin R Shulman GI Caprio S Prediabetes in obese youth: a syndrome of impaired glucose tolerance, severe insulin resistance, and altered myocellular and abdominal fat partitioning Lancet 2003 362 9388 951 957 14511928 10.1016/S0140-6736(03)14364-4 PMC2995523

[B54] Foretz M Hebrard S Leclerc J Zarrinpashneh E Soty M Mithieux G Sakamoto K Andreelli F Viollet B Metformin inhibits hepatic gluconeogenesis in mice independently of the LKB1/AMPK pathway via a decrease in hepatic energy state J Clin Invest 2010 120 7 2355 2369 20577053 10.1172/JCI40671 PMC2898585

[B55] El-Mir M-Y Nogueira V Fontaine E Avéret N Rigoulet M Leverve X Dimethylbiguanide inhibits cell respiration via an indirect effect targeted on the Respiratory Chain Complex I J Biol Chem 2000 275 1 223 228 10617608 10.1074/jbc.275.1.223

[B56] Zhou G Myers R Li Y Chen Y Shen X Fenyk-Melody J Wu M Ventre J Doebber T Fujii N Musi N Hirshman MF Goodyear LJ Moller DE Role of AMP-activated protein kinase in mechanism of metformin action J Clin Invest 2001 108 8 1167 1174 11602624 10.1172/JCI13505 PMC209533

[B57] Pospisilik JA Knauf C Joza N Benit P Orthofer M Cani PD Ebersberger I Nakashima T Sarao R Neely G Esterbauer H Kozlov A Kahn CR Kroemer G Rustin P Burcelin R Penninger JM Targeted deletion of AIF decreases mitochondrial oxidative phosphorylation and protects from obesity and diabetes Cell 2007 131 3 476 491 17981116 10.1016/j.cell.2007.08.047

[B58] Sparks LM Xie H Koza RA Mynatt R Hulver MW Bray GA Smith SR A high-fat diet coordinately downregulates genes required for mitochondrial oxidative phosphorylation in skeletal muscle Diabetes 2005 54 7 1926 1933 15983191 10.2337/diabetes.54.7.1926

[B59] Bonnard C Durand A Peyrol S Chanseaume E Chauvin M-A Morio B xE a Vidal H Rieusset J Mitochondrial dysfunction results from oxidative stress in the skeletal muscle of diet-induced insulin-resistant mice J Clin Invest 2008 118 2 789 800 18188455 10.1172/JCI32601 PMC2176186

[B60] Jans A Sparks LM van Hees AM Gjelstad IM Tierney AC Riserus U Drevon CA Roche HM Schrauwen P Blaak EE Transcriptional metabolic inflexibility in skeletal muscle among individuals with increasing insulin resistance Obesity (Silver Spring) 2011 19 11 2158 2166 21701566 10.1038/oby.2011.149

[B61] Boyle KE Canham JP Consitt LA Zheng D Koves TR Gavin TP Holbert D Neufer PD Ilkayeva O Muoio DM Houmard JA A high-fat diet elicits differential responses in genes coordinating oxidative metabolism in skeletal muscle of lean and obese individuals J Clin Endocrinol Metab 2011 96 3 775 781 21190973 10.1210/jc.2010-2253 PMC3047224

[B62] Keung W Ussher JR Jaswal JS Raubenheimer M Lam VHM Wagg CS Lopaschuk GD Inhibition of Carnitine Palmitoyltransferase-1 activity alleviates insulin resistance in diet-induced obese mice Diabetes 2013 62 3 711 720 23139350 10.2337/db12-0259 PMC3581198

[B63] Muoio Deborah M Noland Robert C Kovalik J-P Seiler Sarah E Davies Michael N DeBalsi KL Ilkayeva Olga R Stevens Robert D Kheterpal I Zhang J Covington Jeffrey D Bajpeyi S Ravussin E Kraus W Koves Timothy R Mynatt Randall L Muscle-specific deletion of carnitine acetyltransferase compromises glucose tolerance and metabolic flexibility Cell Metab 2012 15 5 764 777 22560225 10.1016/j.cmet.2012.04.005 PMC3348515

[B64] Dyck JR Hopkins TA Bonnet S Michelakis ED Young ME Watanabe M Kawase Y Jishage K Lopaschuk GD Absence of malonyl coenzyme A decarboxylase in mice increases cardiac glucose oxidation and protects the heart from ischemic injury Circulation 2006 114 16 1721 1728 17030679 10.1161/CIRCULATIONAHA.106.642009

[B65] Lionetti V Linke A Chandler MP Young ME Penn MS Gupte S d’Agostino C Hintze TH Stanley WC Recchia FA Carnitine palmitoyl transferase-I inhibition prevents ventricular remodeling and delays decompensation in pacing-induced heart failure Cardiovasc Res 2005 66 3 454 461 15914110 10.1016/j.cardiores.2005.02.004

[B66] Muoio DM Way JM Tanner CJ Winegar DA Kliewer SA Houmard JA Kraus WE Dohm GL Peroxisome proliferator-activated receptor-α regulates fatty acid utilization in primary human skeletal muscle cells Diabetes 2002 51 4 901 909 11916905 10.2337/diabetes.51.4.901

[B67] Forman BM Chen J Evans RM Hypolipidemic drugs, polyunsaturated fatty acids, and eicosanoids are ligands for peroxisome proliferator-activated receptors alpha and delta Proc Natl Acad Sci U S A 1997 94 9 4312 4317 9113986 10.1073/pnas.94.9.4312 PMC20719

[B68] Yu K Bayona W Kallen CB Harding HP Ravera CP McMahon G Brown M Lazar MA Differential activation of peroxisome proliferator-activated receptors by eicosanoids J Biol Chem 1995 270 41 23975 23983 7592593 10.1074/jbc.270.41.23975

